# Associations between outpatient care and later hospital admissions for patients with chronic obstructive pulmonary disease - a registry study from Norway

**DOI:** 10.1186/s12913-024-10975-4

**Published:** 2024-04-22

**Authors:** Tron Anders Moger, Jon Helgheim Holte, Olav Amundsen, Silje Bjørnsen Haavaag, Anne Edvardsen, Line Kildal Bragstad, Ragnhild Hellesø, Trond Tjerbo, Nina Køpke Vøllestad

**Affiliations:** 1 Department of Health Management and Health Economics, Institute of Health and Society, University of Oslo, Oslo, Norway; 2 Department for Interdisciplinary Health Sciences, Institute of Health and Society, University of Oslo, Oslo, Norway; 3 Department of Public Health Science, Institute of Health and Society, University of Oslo, Oslo, Norway; 4 Department of Pulmonary Medicine, Akershus University Hospital, Lørenskog, Norway; 5 Faculty of Health Sciences, Department of Rehabilitation Science and Health Technology, Oslo Metropolitan University, Oslo, Norway

**Keywords:** COPD, Registry data, Outpatient care, Hospital admissions, Readmissions

## Abstract

**Background:**

Although chronic obstructive pulmonary disease (COPD) admissions put a substantial burden on hospitals, most of the patients’ contacts with health services are in outpatient care. Traditionally, outpatient care has been difficult to capture in population-based samples. In this study we describe outpatient service use in COPD patients and assess associations between outpatient care (contact frequency and specific factors) and next-year COPD hospital admissions or 90-day readmissions.

**Methods:**

Patients over 40 years of age residing in Oslo or Trondheim at the time of contact in the period 2009–2018 were identified from the Norwegian Patient Registry (in- and outpatient hospital contacts, rehabilitation) and the KUHR registry (contacts with GPs, contract specialists and physiotherapists). These were linked to the Regular General Practitioner registry (characteristics of the GP practice), long-term care data (home and institutional care, need for assistance), socioeconomic and–demographic data from Statistics Norway and the Cause of Death registry. Negative binomial models were applied to study associations between combinations of outpatient care, specific care factors and next-year COPD hospital admissions and 90-day readmissions. The sample consisted of 24,074 individuals.

**Results:**

A large variation in the frequency and combination of outpatient service use for respiratory diagnoses (GP, emergency room, physiotherapy, contract specialist and outpatient hospital contacts) was apparent. GP and outpatient hospital contact frequency were strongly associated to an increased number of next-year hospital admissions (1.2–3.2 times higher by increasing GP frequency when no outpatient hospital contacts, 2.4-5 times higher in combination with outpatient hospital contacts). Adjusted for healthcare use, comorbidities and sociodemographics, outpatient care factors associated with lower numbers of next-year hospitalisations were fees indicating interaction between providers (7% reduction), spirometry with GP or specialist (7%), continuity of care with GP (15%), and GP follow-up (8%) or rehabilitation (18%) within 30 days vs. later following any current year hospitalisations. For 90-day readmissions results were less evident, and most variables were non-significant.

**Conclusion:**

As increased use of outpatient care was strongly associated with future hospitalisations, this further stresses the need for good communication between providers when coordinating care for COPD patients. The results indicated possible benefits of care continuity within and interaction between providers.

**Supplementary Information:**

The online version contains supplementary material available at 10.1186/s12913-024-10975-4.

## Background

Chronic obstructive pulmonary disease (COPD) is a heterogeneous lung condition characterised by chronic respiratory symptoms (such as dyspnea, cough and activity limitation) due to abnormalities in the airways and lungs. COPD causes persistent, often progressive, airflow obstruction (Global Initiative for Chronic Obstructive Lung Disease [1)]. COPD is currently the third leading cause of death worldwide, with more than 3 million deaths and around 390 million cases in 2019 [1, 2]. The prevalence and burden of COPD is expected to increase in the near future due to continued exposure to risk factors and aging of the populations worldwide. This will result in more people expressing the long-term effects of exposure to the COPD risk factors [1, 3, 4]. COPD is commonly considered among conditions where appropriate primary/outpatient care can reduce the risk of hospitalisations [5, 6]. In Germany, it has been estimated that 76% of COPD hospital admissions are avoidable [7]. Hospitalisation is the most important direct cost for COPD patients [8]. One important aim of disease management is to prevent exacerbations, which are episodes of acute symptom worsening frequently resulting in hospitalisation. Exacerbations have considerable negative impact on patients’ quality of life and are the leading cause of healthcare utilisation and costs in COPD care [3].

Risk factors for exacerbations, hospital admissions and readmissions include smoking, age, duration and severity of COPD, health status, socioeconomic status, various comorbidities (e.g. diabetes, heart failure, renal failure, depression, alcohol use, hypertension), various clinical indicators (e.g. blood-gas levels, biomarkers) and previous exacerbations and hospitalisations [9–14]. The heterogeneous nature and long duration of COPD, along with presence of comorbidities, mean that healthcare providers at all levels are involved in the patient follow-up and treatment [3]. Many patients need to navigate in a highly complex web of care [6, 15].

However, it is generally challenging to identify care over time at the individual level due to insufficient or unavailable data. The scarcity of studies including healthcare contacts across providers [16], or studies focusing on system level variables (e.g. care type, general practitioner (GP) visit frequency) [17] may be related to these difficulties. Hence, there is a lack of literature describing the frequency and variation in service use by types and combinations of care providers, and their association with hospital admissions and readmissions. In Norway it is possible to link data from health registries covering both primary and specialist care since 2008 at the individual level.

This paper has an exploratory focus. The first aim is to describe in- and outpatient contacts in a population-based COPD sample by provider type and contact frequency per year. Second, we study the association between combinations of outpatient providers involved in the follow-up (GP, physiotherapist, specialist, and outpatient hospital) and next-year COPD hospital admissions. We examine the extent to which increased contact with outpatient providers is associated with a higher number of COPD hospital admissions, even after adjusting for comorbidities, socioeconomic, and–demographic factors. Third, we assess associations between specific factors in the outpatient care (e.g. interaction between providers, continuity of care with the GP, relevant follow-up of the patient stated in COPD guidelines) and (1) next-year COPD hospital admissions and (2) 90-day readmissions. By assuming that the COPD health status of the patient is reflected in yearly healthcare use and comorbidities, these analyses may identify factors associated with beneficial effects on either of the outcomes. As appropriate outpatient care could reduce COPD hospital admissions, analysis (1) will be of particular interest to providers in the outpatient services by describing outpatient care factors associated with higher/lower risk of hospital admissions. Analysis (2) is of particular interest to providers in the inpatient services, describing outpatient care factors associated with higher/lower risk of readmissions.

## Methods

### Design and setting

This a retrospective exploratory quantitative registry-based study conducted as part of the project Innovations in use Of REGistry data (INOREG) at the Institute of Health and Society, University of Oslo. Data on COPD patients from several national registries covering in- and outpatient care, socioeconomics and -demographics were linked at the individual level in the project. By collaboration with the Department of Health in Oslo and Trondheim municipalities, it was also possible to access long term care data (home services and institutional stays). Oslo is the most populous, while Trondheim is the third most populous municipality in Norway. Hence, the use of health services at all levels is registered for each patient over time. For more information about the project, see [18].

In Norway, the financing of specialist care is executed by the central state and provided by four regional health authorities. While inpatient care is free of charge for the patient, outpatient care and contract specialist visits have an out-of-pocket fee of approximately 33€ [19]. Contract specialists are specialists on contract with a regional health authority. The number of fully private specialists is limited in Norway and not available in registries. Provision of primary (including long-term) care is a municipal responsibility. Citizens in Norway are entitled to enlistment with a general practice or a named regular GP. The GP has a role as a gatekeeper, referring patients to contract specialists, outpatient hospital and physiotherapists (referral mandatory until 2019). GPs and physiotherapists are mainly independent contractors with the municipality, and not municipal employees. Municipalities also operate primary care emergency rooms, which can be contacted without appointment and after hours. The out-of-pocket fees in primary care vary from around 15€ to 25€ depending on the service [19]. However, for the individual patient there is a ceiling for the total out-of-pocket fees of roughly 230€ per year in most outpatient services [19].

### Sample selection and data sources

Patients over 40 years of age, residing in Oslo or Trondheim at the time of first contact with a primary diagnosis of COPD (ICD-10: J43, J44, ICPC-2: R95) during 2009–2018 were identified from the Norwegian Patient Registry (NPR, in- and outpatient treatment, private rehabilitation) and Control and Payment of Reimbursement to Health Service Providers (KUHR, contacts with GPs, contract specialists and physiotherapists). At least one year of follow-up from the first healthcare contact with COPD as main diagnosis was required. The sample was further linked to data from the Regular General Practitioner registry (characteristics of the regular GP and practice), municipality electronic patient journal (MEPJ, services such as home nursing, long-term care, need for assistance by activities of daily living), socioeconomic and–demographic data from Statistics Norway and time of death from the Cause of Death Registry.

Data from all sources was available also for the years 2008 and 2019. Hence, for patients diagnosed at an unknown time prior to 2009, we captured their health status when entering the analyses using data for 2008. This included possible healthcare contacts with COPD or respiratory diagnoses, to analyse all patients hospitalised in 2009. Similarly, we analysed hospitalisations for patients first diagnosed in 2018 using data for 2019. Hence, the sample should be representative of the general population of COPD patients from 2009 to 2018. 24,074 individuals were included in the sample.

### Main outcomes

The two main outcomes were next-year COPD hospital admissions and 90-day COPD readmissions. Hospital admissions were counted per year. If discharge and admission dates were less than a day apart, the new admission was not counted. Analyses were performed separately for hospital admissions and 90-day readmissions with COPD (ICD-10: J43, J44) as the main diagnosis.

### Independent variables

The independent variables are shown in Table 1. To separate between COPD-related and other care, outpatient care was split into contacts with respiratory (primary or first secondary diagnosis ICD-10: J, ICPC-2: R) and non-respiratory diagnoses. In primary care, the provider has to register a primary diagnosis in order to get reimbursed, hence the vast majority of contacts in KUHR only include one diagnosis. Rehabilitation includes out- and inpatient rehabilitation in hospital, rehabilitation in private clinics on contract with the regional health authority, and home-based and short-term institution-based rehabilitation from the municipality. The content of the rehabilitation is not known. Similarly, outpatient contact with hospital excludes those where the primary focus is rehabilitation (ICD-10 code Z50). Although the data are comprehensive, a weakness is that clinical information on COPD severity is not available in the administrative registry data. However, we assume that the frequency of healthcare contacts with a respiratory main diagnosis reflect the COPD health status. Correspondingly, the frequency of contacts with non-respiratory diagnoses (including non-COPD hospital admissions) and comorbidities should be correlated to the overall health status of the patient.

In addition to variables related to frequency of healthcare contacts, a number of outpatient care indicators were constructed (Table 1). Continuity in primary care has been shown to be associated with lower hospitalisation rates [20, 21], including some studies focusing specifically on COPD [22–24]. Continuity was measured with the Bice-Boxerman continuity of care index (COCI) [25]. The Norwegian COPD guidelines [26] recommends check-ups with GP once per year for patients with stable mild to moderate COPD, twice per year for patients with stable severe COPD. The check-ups should include spirometry, and patients should be referred to physiotherapists for training if their dyspnoea scale is of grade 2 or higher, as per the modified Medical Research Council Dyspnoea Scale [27]. Hospitalisations with COPD should be followed by a check-up with the GP within four weeks in cases without respiratory failure, otherwise follow-up is done by the hospital [26]. If in need of rehabilitation, this should be initiated shortly (not specified) after discharge. Care interaction in a specific patient contact was constructed from interaction fee codes in KUHR. The exact content in the interaction is unknown. As alternative measures of care interaction, we constructed variables capturing the propensity of the GP to refer/involve other providers in the treatment of their COPD patients in the sample. These were calculated from the GPs contact rate (sum of number of contacts divided by sum of follow-up years for the patients on their list in the sample) to respectively contract specialists, physiotherapists and outpatient hospital. The former care interaction variable is thus patient specific, whereas the latter variables are more related to the practice of the GP. Associations to general characteristics of the GP, such as a specialty in general practice (better educated), being in a group practice (interact with colleagues) and patient list length (time for each patient), were also included.

**Table 1:** List of independent variables used in the analyses. NPR = Norwegian Patient Registry, KUHR = Control and Payment of Reimbursement to Health Service Providers, RGPR = Regular General Practitioner registry, MEPJ = municipality electronic patient journal

Variable	Definition	Source
**Frequency of outpatient care per year, respiratory diagnoses**		
Contact with GP, respiratory diagnoses	Number of contacts with respiratory diagnoses	KUHR
Emergency room contacts, respiratory diagnoses	Number of contacts with municipal emergency room	KUHR
Contact with contract specialist, respiratory diagnoses	Number of contacts	KUHR
Contact with physiotherapist, respiratory diagnoses	Number of contacts with general physiotherapist, manual physiotherapist, occupational therapist	KUHR
Outpatient contact with hospital, respiratory diagnoses	Number of contacts	NPR
Rehabilitation, respiratory diagnoses	Number of out- and inpatient rehabilitation contacts in hospital and private clinics	NPR
**Frequency of healthcare per year, non-respiratory diagnoses**		
Contact with GP, non-respiratory diagnoses	Number of contacts with non-respiratory diagnoses	KUHR
Emergency room contacts, non-respiratory diagnoses	Number of contacts with municipal emergency room	KUHR
Contact with contract specialist, non-respiratory diagnoses	Number of contacts	KUHR
Contact with physiotherapist, non-respiratory diagnoses	Number of contacts with general physiotherapist, manual physiotherapist, occupational therapist	KUHR
Outpatient contact with hospital, non-respiratory diagnoses	Number of contacts	NPR
Rehabilitation, non-respiratory diagnoses	Number of rehabilitation contacts, including home-based and short-term rehabilitation from the municipality	NPR/MEPJ
Hospital admissions, non-respiratory diagnoses	Number of hospital admissions with non-respiratory diagnoses	NPR
**Outpatient care indicators per year**		
Continuity of GP care	Continuity of care index (COCI) for GP contact dispersion on scale 0–1. Higher values imply contact with fewer unique GPs, thus better continuity.	KUHR
Spirometry	Indicator of spirometry performed at least once at GP or contract specialist during the year	KUHR
Early rehabilitation, respiratory diagnoses	Three categories: Rehabilitation within 30 days following at least one COPD hospital admission; only later rehabilitation (up to 6 months) after COPD hospital admissions; no COPD hospital admissions or admissions with rehabilitation during the year	NPR/MEPJ
Early follow-up by GP, respiratory diagnoses	Three categories: Contact with GP within 30 days after at least one COPD hospital admission; no or later follow-up only; no COPD hospital admissions during year	NPR/MEPJ
Care interaction, respiratory diagnoses	Indicator of fees for interaction between either GP/specialist, GP/long-term care or GP/physiotherapist at least once during year	KUHR
Home visits by GP or physiotherapist, respiratory diagnoses	Number of home visits by GP or physiotherapist performed during the year	KUHR
**Characteristics of the regular GP**		
GP specialist	Indicator of GP being a specialist in general practice	RGPR
GP group practice	Indicator of GP practicing in a location shared with other GPs	RGPR
List length	Number of individuals on the GP’s patient list	RGPR
Contact rate GP and contract specialist, respiratory diagnoses	Contract specialist contacts per year per listed patient in sample	KUHR/RGPR
Contact rate GP and physiotherapist, respiratory diagnoses	Physiotherapist contacts per year per listed patient in sample	KUHR/RGPR
Contact rate GP and outpatient hospital, respiratory diagnoses	Outpatient hospital contacts per year per listed patient in sample	NPR/RGPR
**Other health factors**		
Comorbidities	Yearly indicators for each of 17 comorbidities commonly associated with COPD, see Table S1 for details	NPR/KUHR
Need-score for assistance in daily living	Yearly sum score of items in the activities of daily living (ADL) checklist at start of follow-up year, assume score of 0 if not receiving any long-term care service	MEPJ
Home nursing	Yearly indicator of receiving home nursing	MEPJ
Nursing home	Yearly indicator of being in nursing home	MEPJ
Death	Yearly death indicator yes/no to capture deteriorating health near end of life	Cause of Death Registry
**Socioeconomics and -demographics**		
Updated yearly	Age and gross income	Statistics Norway
Constant	Gender, marital status when entering sample, highest attained education, indicator of permanent disability pension prior to or during follow-up	Statistics Norway

### Statistical methods

Descriptives on socioeconomics and -demographics and long-term care for the sample are presented as means and standard deviations for the continuous variables, and as percentages and numbers for the categorical variables. For in- and outpatient care and the care indicators (where relevant) we present the total number, the percent and number of person-years with at least one occurrence of the service, the mean number per patient per follow-up year and the 1st and 3rd quartile of the distribution. A mean value above the 3rd quartile and a low percentage of person-years with at least one occurrence imply both that the service is rare in the sample and that there is a high number of contacts in some patients or follow-up years.

The yearly hospital admissions and 90-day readmissions are count outcomes with skewed distributions (there were 372 observations with more than one readmission within 90 days). Hence, negative binomial GLM models reporting incidence rate ratios (IRR) were applied to study the associations between the variables in Table 1 and next-year number of COPD hospital admissions and 90-day readmissions.

In the regression analyses of next-year hospital admissions, the data were structured per year of follow-up from first COPD registration for each patient. We studied how combinations of outpatient service use in the current year was associated to next-year hospital admissions. The use of services with a respiratory diagnosis were further categorised as follows. First, GP contacts were categorised into no contacts, 1–2 contacts, 3–4 or 5 or more contacts in a year. Contract specialist, physiotherapy and outpatient hospital contacts were categorised in no contact/at least one contact. Second, we constructed a variable containing all combinations of the categorised service variables, thus capturing how frequent the services were used in combination per year. We studied whether use of more outpatient services was consistently associated with a higher number of next-year COPD hospital admissions. To the contrary, if e.g. contact with contract specialists was associated with a reduced number of next-year hospital admissions within a given combination of GP, physiotherapy and outpatient hospital contacts, it could indicate a beneficial effect of access to specialist. For the care indicators and GP characteristics listed in Table 1, similar arguments can be made. Assume that patients within each combination of outpatient respiratory healthcare use are relatively homogenous in their COPD health status. A variable associated with reduced numbers of next-year hospital admissions could then indicate a beneficial effect. Variables for the number of non-respiratory outpatient contacts were entered as continuous variables in the models. Prior to the categorisation described above, the correlation between all in- and outpatient frequency variables was checked using Spearman’s Rho. These were all surprisingly low (below 0.35), thus showing little indication that high use of one health service during a year implied high use of other services as well. We also included current year COPD hospital admissions in the analysis to adjust for the large variation in admissions both between and within patients over time. Thus we estimated effects of the independent variables conditional on the number of current year hospital admissions.

In the regression analyses of the number of 90-day readmissions, the data were structured per index COPD hospital admission, defined as admissions with no COPD hospitalisation in the previous 90 days. This was done to avoid overlap between periods. The number of outpatient contacts and non-COPD hospital admissions were counted in the 90 days prior to each index hospital admission (instead of during the year). Hence, we analysed whether outpatient service use in the period directly preceding the index admission was associated with higher/lower risk of readmissions. The indicators of GP follow-up and rehabilitation within 30 days (Table 1) were defined with respect to each index admission, with late rehabilitation occurring between 30 and 90 days after discharge. Due to the smaller sample size, variables for respiratory outpatient contacts were entered individually as continuous variables and not as combinations in the models.

To account for repeated measurements of individuals over time, standard errors were clustered by patient-id in the analyses of both outcomes. Most independent variables were time-dependent (except gender), and as such updated yearly in the analysis (or in 90-day intervals in the case of outpatient care contacts in the analysis of readmissions). This was assumed to reflect deterioration in the patients’ health over time. In the analyses, it was captured by the variables for comorbidities, respiratory and non-respiratory healthcare use, and age. Death or, in rare cases, emigration, lead to incomplete observation of the most recent follow-up year for a patient. We pragmatically included an indicator for next-year/90-day death in the models to capture deteriorating health near the end of life, as it could be associated with an additional increase in admissions. We present adjusted regression results for the combinations of outpatient service use, care indicators, characteristics of the GP, comorbidities mentioned in the COPD guidelines [26] and socioeconomics in the main text. Adjusted results for the remaining variables, as well as unadjusted results for all variables, are presented in the Supplementary Material. The data were analysed in Stata Version 16.1, and a 5% significance level was applied throughout.

**Table 2:** Descriptive statistics for the sample of COPD patients. SD = standard deviation

Main descriptives				
No. of patients	24,074			
No. of person-years	148,128			
Follow-up years per patient, mean (SD)	6.2 (3.0)			
Age at inclusion, mean (SD)	65 (12)			
Females (n)	47% (11,295)			
Number of comorbidities, mean (SD)	4.0 (2.3)			
Receiving disability pension prior to or during follow-up (n)	35% (8,533)			
Receiving home nursing during follow-up (n)	28% (6,825)			
Admitted to nursing home during follow-up (n)	6% (1,344)			
Dead within follow-up (n)	27% (6,521)			
No. patients with at least one COPD hospital admission during follow-up	8,829			
No. person-years with at least one COPD hospital admission during follow-up	58,193			
**Inpatient care**	**Total**	**% person-years with > 0 occurrences (n)**	**Mean per patient per year**	**1st -3rd quartile**
COPD hospital admissions, total sample	25,012	10.5% (15,497)	0.2	0.0-0.2
-Among patients with COPD hospital admissions during follow-up only	25,012	26.6% (15,497)	0.6	0.1–0.6
Non-COPD hospital admissions	80,601	27.8% (41,152)	0.6	0.0-0.7
-Among patients with COPD hospital admissions during follow-up only	42,670	34.8% (20,263)	0.8	0.2-1.0
**Readmissions**:				
No. of COPD index admissions used in analysis	18,372			
No outpatient respiratory care during year prior to index admission (n)	11.1% (2,046)			
90-day COPD readmissions	2,642	10.8% (2,095) of index admissions	0.14 per admission	0.0–0.0 per admission
**Outpatient care, respiratory diagnoses**	**Total**	**% person-years with > 0 occurrences (n)**	**Mean per patient per year**	**1st -3rd quartile**
GP contacts	485,121	67.3% (99,714)	3.3	1.0-4.3
Emergency room contacts	26,388	9.9% (14,636)	0.1	0.0–0.0
Physiotherapy contacts	292,104	6.6% (9,717)	1.6	0.0–0.0
Contract specialist contacts	42,679	13.6% (20,174)	0.3	0.0-0.2
Outpatient hospital contacts	64,709	17.2% (25,512)	0.4	0.0-0.4
Rehabilitation	12,297	2.2% (3,255)	0.1	0.0–0.0
**Outpatient care, non-respiratory diagnoses**				
GP contacts	1,488,029	90.5% (134,066)	10.4	4.3–13.7
Emergency room contacts	67,217	19.4% (28,753)	0.7	0.0-0.7
Physiotherapy contacts	539,714	18.2% (26,964)	0.2	0.0-0.1
Contract specialist contacts,	196,774	42.2% (62,552)	1.3	0.0-1.7
Outpatient hospital contacts	371,300	54.6% (80,828)	2.7	0.5–3.3
Rehabilitation	35,111	7.4% (11,013)	0.3	0.0-0.1
**Care indicators**				
Continuity of GP care	N/A	N/A	0.7	0.6–0.9
Spirometry performed at GP or contract specialist	34,440	23.3% (34,440)	0.2	0.0-0.4
Home visits by GP or physiotherapist	21,972	6.2% (9,198)	0.2	0.0-0.1
Care interaction fees	32,503	9.2% (13,564)	0.3	0.0-0.2

## Results

Descriptive statistics for the sample are shown in Table 2. The analyses included 148,128 person-year observations from 24,074 patients. The patients had a mean age of 65 years (SD = 12 years) with slightly more males than females. Home nursing was relatively frequent in the sample, and 28% were receiving this either already when entering the follow-up period, or during follow-up. There was at least one COPD hospital admission in 10.5% of the yearly observations, and around one-third of the patients had one or more COPD hospital admissions during follow-up (8,829 patients). Some patients had spells with frequent COPD admissions. This is seen from the large discrepancy between the number of COPD index admissions used in readmission analysis (18,372) and the total number of hospital admissions (25,012), and the relatively low percentage of person-years with COPD admissions among patients hospitalised at least once during follow-up (26.6%). In addition, the mean number of COPD admissions among patients hospitalised at least once was at the level of the 3rd quartile of the distribution (0.6 per patient per year). Interestingly, 11.1% of the COPD index admissions had no indications of outpatient respiratory healthcare the year before the admission. There was an average of 0.14 90-day COPD readmissions per index admission (10.8% of the index admissions had at least one readmission). Some of the outpatient services showed a particularly skewed distribution, with the mean above or at the 3rd quartile. This applied to physiotherapy contacts, emergency room contacts, hospital admissions, rehabilitation, and outpatient hospital/contract specialist contacts for respiratory diagnoses. For most services, the between- and within-patient variance in contact frequency per follow-up year were of similar magnitude (not shown).

**Fig. 1:**
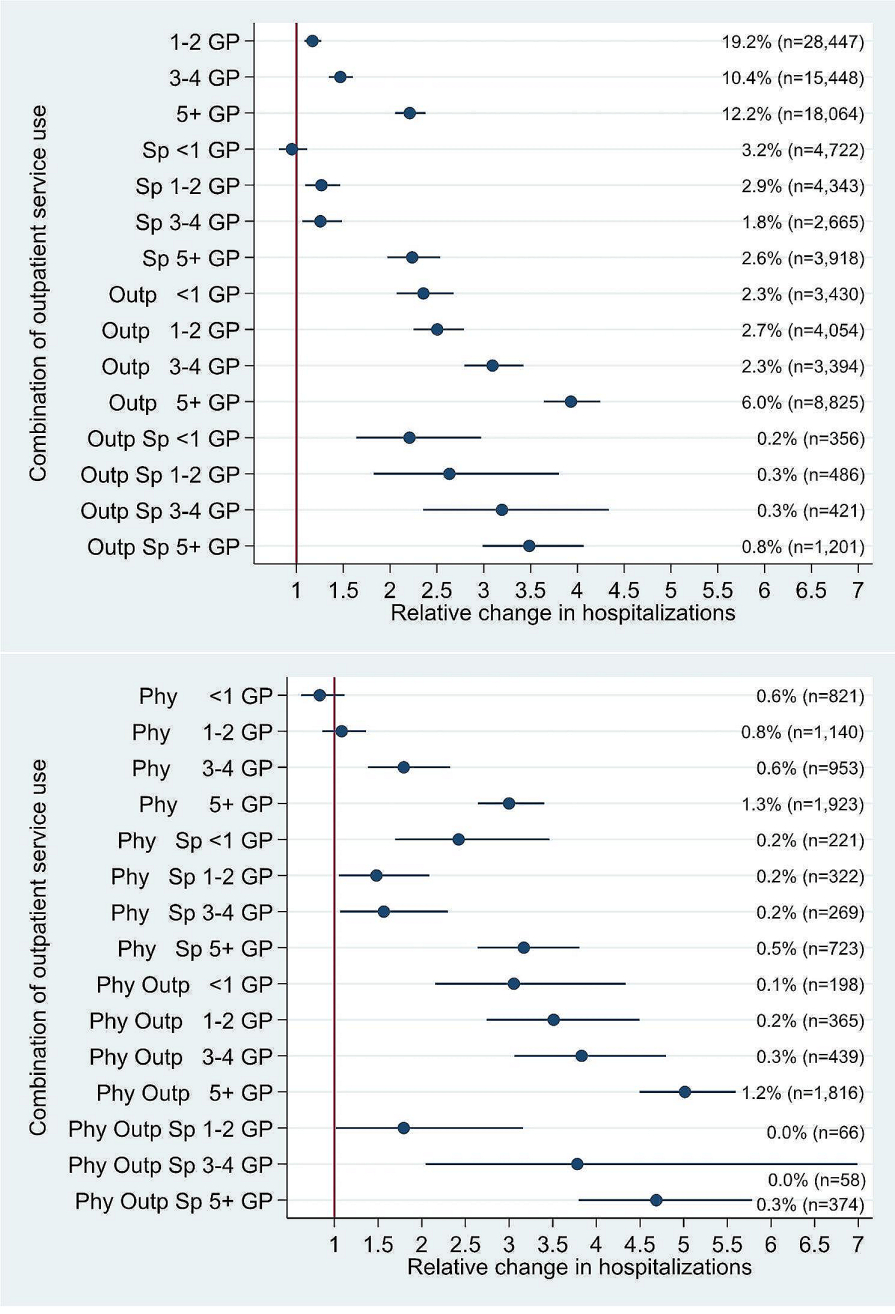
Relative change (incidence rate ratio and 95% confidence interval) in next-year COPD admissions for combinations of current year health service use, % of total and number of observations within each combination Note: Adjusted for variables in Table 3 and Table S2. The reference category is no contacts for respiratory diagnoses (26% of total, *n* = 38,637 observations). The category no GP contact and use of all other services is omitted due to few observations (*n* = 29). *N* = 148,128 observations from 24,074 patients. GP = general practitioner, Sp = contract specialist contact, Outp = outpatient contact with hospital, Phy = physiotherapy contact

### Regression results, COPD hospital admissions

Figure 1 shows the results for the combination of outpatient services for respiratory diagnoses in the adjusted model with number of COPD hospital admissions the following year as the outcome. Unadjusted results are presented in Figure S1. The results were stable between the adjusted and unadjusted models. Due to the high variation in health service use also within patients over time, patients may switch combination from one year to the next. Having 1–2 GP contacts in a year constituted around one-third of the observations where at least one service was used. Both contract specialist and outpatient contacts with hospital were relatively frequent, also without having a GP contact in the same year. Involving a physiotherapist was less frequent. A higher frequency of GP contacts was generally associated with more next-year COPD hospital admissions, except for combinations involving outpatient hospital and physiotherapy or contract specialist contacts. However, the data was scarce for some of the combinations. There was a particular increase for combinations including outpatient contacts with hospital. There were no indications that the use of a service was associated with fewer next-year hospital admissions. Within specific combinations of three out of the four services, the presence or absence of any fourth service did reduce the IRR. For instance, having physiotherapy contacts vs. not seemed consistently associated with the same number or relatively more nextyear hospital admissions, regardless of the combination of the other three services.

Table 3 shows the adjusted regression results for the care indicators and characteristics of the GP, socioeconomics and selected comorbidities. The results for the remaining variables in the model are shown in Table S2, while unadjusted results are given in Tables S3 and S4. When adjusting for health service use (Fig. 1) and additional variables (Table S2), some of the care indicators were associated with a reduction in next-year hospital admissions. This applied to higher continuity with the GP (IRR = 0.85), having spirometry measurements by GP or contract specialist (IRR = 0.93), use of fees indicating interaction between outpatient providers (IRR = 0.93), and having early vs. late rehabilitation (IRR = 0.82) or follow-up by GP (IRR = 0.92) after at least one current year COPD hospital admission. Around 90% of the spirometry registrations were by GPs, the remainder by contract specialists. There were no substantial associations for variables describing characteristics of the GP, except that having a GP with higher contact rate to contract specialist was associated with more next-year hospital admissions. Although the estimates in Table 3 were modest compared to those for health service use in Fig. 1, they were of similar magnitude as many socioeconomic, -demographic and comorbidity estimates in Table S2. The associations found for most of the latter variables were relatively stable between the unadjusted and adjusted models, but the IRRs were attenuated. Nine of 17 comorbidities were associated with an increased number of next-year hospital admissions after adjustment, the highest estimates being for alcoholism (IRR = 1.45), depression (IRR = 1.20) and osteoporosis (IRR = 1.14). Being on permanent disability pension or being single was also associated with higher numbers of next-year hospital admissions (Table S2), while higher income and education were associated with lower numbers (Table 3).

### Regression results, 90-day COPD readmissions

In the analysis of readmissions on the other hand, few of the variables on outpatient healthcare, care indicators and GP characteristics reached statistical significance in the adjusted analysis (Table 3 and S2). Hospital admissions for other diagnoses (IRR = 1.17), increasing GP contact frequency (IRRs = 1.30–1.40) and municipal emergency room contacts (IRR = 1.19) with respiratory diagnoses in the prior 90 days were associated with more 90-day readmissions. Contract specialist contacts for any diagnosis were associated with fewer readmissions (IRRs 0.78 and 0.91). None of the care indicators, GP characteristics, comorbidities (except being underweight) and socioeconomic variables were significant in the adjusted analysis. A majority of the variables had IRRs closer to 1 also in the unadjusted analyses (Tables S3 and S4).

**Table 3:** Regression results for the outpatient care indicators, characteristics of the regular GP, and selected socioeconomic and comorbidity variables. Adjusted for variables in Fig. 1 (next-year COPD admissions) and Table S2. IRR = Incidence rate ratio, 95%-CI = 95% confidence interval. *=significant at 5%-level

Outcome:	Next-year COPD hospital admissions (n = 148,128)	90-day readmissions (n = 18,372 index hospital admissions)
Independent variable:	IRR (95%-CI)	IRR (95%-CI)
**Care indicators**		
Spirometry performed at GP or contract specialist	0.93 (0.88–0.97)*	1.04 (0.91–1.20)
Continuity within GP care	0.85 (0.79–0.91)*	0.87 (0.74–1.04)
Rehabilitation within 30 days vs. later after COPD hospital admission	0.82 (0.70–0.95)*	0.86 (0.70–1.07)
No hospital admission/rehabilitation vs. rehabilitation after COPD hospital admission	0.94 (0.82–1.07)	0.43 (0.37–0.50)*
Follow-up by GP within 30 days vs. no/later follow-up after COPD hospital admission	0.92 (0.87–0.98)*	1.09 (0.97–1.22)
No hospital admissions vs. no/later follow-up by GP after COPD hospital admission	0.45 (0.42–0.48)*	N/A
Care interaction fees	0.93 (0.86–0.99)*	1.01 (0.87–1.16)
Home visits by GP or physiotherapist	1.00 (1.00-1.01)	1.01 (1.00-1.02)
**Characteristics of the regular GP**		
GP specialist	0.99 (0.94–1.05)	1.01 (0.87–1.15)
GP group practice	0.91 (0.81–1.03)	1.05 (0.80–1.39)
List length per 100 patients	0.99 (0.98-1.00)*	1.00 (0.98–1.01)
Contact rate GP and contract specialist, respiratory diagnoses	1.08 (1.03–1.13)*	1.08 (0.99–1.19)
Contact rate GP and physiotherapist, respiratory diagnoses	1.00 (1.00–1.00)	1.00 (0.99–1.01)
Contact rate GP and outpatient hospital, respiratory diagnoses	1.05 (0.99–1.11)	1.01 (0.98–1.04)
**Comorbidities**		
Alcoholism	1.45 (1.31–1.60)*	1.20 (0.94–1.52)
Anemia	0.95 (0.87–1.05)	1.16 (0.96–1.40)
Depression	1.20 (1.12–1.28)*	1.00 (0.87–1.15)
Diabetes	0.91 (0.85–0.98)*	0.89 (0.75–1.05)
Lung cancer	1.09 (0.97–1.23)	1.14 (0.90–1.44)
Mental disorders	1.02 (0.91–1.14)	0.96 (0.72–1.27)
Osteoporosis	1.14 (1.05–1.22)*	1.13 (0.96–1.34)
Underweight	1.03 (0.97–1.10)	1.21 (1.03–1.42)*
**Socioeconomics**		
Gross income (per 100,000 NOK)	0.96 (0.93–0.99)*	0.98 (0.94–1.02)
Education: Secondary vs. primary	0.89 (0.85–0.93)*	0.96 (0.86–1.07)
College/university vs. primary	0.69 (0.63–0.74)*	0.94 (0.79–1.13)
Permanent disability pension	1.36 (1.29–1.44)*	1.02 (0.89–1.18)

## Discussion

### Main findings

Not surprisingly, there was considerable variation in the use of services across and within patients over time. However, the results also showed that in most person-years patients had up to two GP contacts per year and did not use other services (Fig. 1). It was clear that the previous use of outpatient services for respiratory diagnoses, particularly by increasing number of GP contacts and outpatient contacts with hospital, was strongly associated to future COPD hospital admissions. There were also indications that some of the specific factors in the outpatient care could be associated with a reduced number of next-year hospital admissions. For 90-day readmissions, on the other hand, the associations to outpatient services were less clear. The associations found for socioeconomic and–demographic variables, previous hospitalisations and continuity of care with GP are generally in line with previous reviews [9–12] and studies [23–25].

The literature has lacked a detailed description of healthcare utilization in a general sample of COPD patients, which we aim to address in this study. This also means that few studies have assessed the association between outpatient healthcare use and later hospital admissions. Describing outpatient care and studying its associations with hospitalisation is particularly important for chronic conditions such as COPD. There is an assumption that appropriate outpatient care may reduce the risk of exacerbations. We have only found two studies utilizing claims data focused on this association [28, 29]. However, due to differences in study design and the fact that healthcare service use is analysed at the patient level over several years in our analysis, the results are difficult to compare. Previous analyses of readmissions [13, 17, 30, 31] have lacked the inclusion of variables related to outpatient healthcare use. Additionally, these studies have primarily focused on all-cause readmissions, rather than specifically examining COPD readmissions.

### Extent of outpatient respiratory care and associations to hospital admissions

The strong associations between combinations of outpatient services for respiratory diagnoses and later COPD hospital admissions suggests that data on outpatient care should be included when analysing risk of hospitalisation for COPD patients in the absence of clinical severity information, both as important risk factors and to improve predictive models. The fact that predictive models for hospitalisation and emergency department visits should be built on outpatient data, mentioning primary care data as the most applicable, has also been highlighted in a general setting [32]. From marginal effects estimated by the negative binomial model, an IRR of 2 in Fig. 1 corresponds to around 0.18 additional hospitalisations per person-year, twice the average observed for the total sample in Table 2, or around 30% higher than observed for patients having COPD admissions during follow-up. Several of the combinations with an IRR larger than 2 are relatively frequent in the sample, such as seeing the GP five or more times during a year or combinations involving GP and outpatient hospital. A significant proportion of high-risk patients may thus be identified during outpatient contact with hospital. On the other hand, in around 11% of the index hospital admissions there were no respiratory outpatient care during the previous year, and a significant proportion of undiagnosed COPD cases has been documented, also in Norway [33]. These findings indicate that in many cases it could be difficult to capture patients and significantly reduce the risk of future hospital admissions.

### Effects of outpatient care indicators

We found indications of reductions in hospital admissions when having greater continuity with the GP, having a spirometry measurement at GP or contract specialist, use of fees indicating interaction between outpatient providers and early vs. later rehabilitation following hospital admissions. It is unlikely that performing spirometry at the GP in itself should reduce the number of future hospital admissions, but it could indicate more focused outpatient COPD follow-up. An alternative explanation could be that such tests are more frequently needed in early phases to assess the functional impairment. Also, use of interaction fees could indicate more active treatment. Still, it is difficult to assess if these interpretations are correct. Prior to adjustment for health service use and comorbidities, the care indicators (spirometry, care interaction fees, home visits, early follow-up by GP) were either non-significant or associated with increased numbers of next-year hospital admissions. The reduction in IRR after adjustment is expected, as without adjustment for outpatient contacts and comorbidities the COPD and general health status of each patient during a year will to a larger extent be captured by the care indicator variables. Adjusting for the frequency and type of providers involved in the outpatient care is assumed to add information on the health status, and hence reduce bias in estimates for the effects of the care indicator variables.

### Characteristics of the regular GP

The associations to increased number of next-year hospital admissions found for the GP’s contact rate to contract specialist and outpatient hospital, even after adjustment for health service use and comorbidities, could be a result of some GPs having more severely ill patients on their list, or alternatively being less confident in the follow-up of COPD patients [34, 35].

### Analysis of readmissions

The lack of significant findings in the adjusted analysis of 90-day readmissions could be related to the smaller sample size. However, the IRRs of most independent variables are either closer to no association also in the unadjusted analyses or change relatively less from the unadjusted to the adjusted analyses, compared to the results for COPD admissions. This could indicate that the outpatient variables considered here do not capture factors associated with readmissions as well as for COPD admissions in general. The increased number of readmissions observed when having at least one GP contact or municipal emergency room contacts during the previous 90 days could signify that these cases are more complex, perhaps with less consistent prior follow-up of the COPD. The reduction in number of readmissions observed when having prior contract specialist contact could indicate the opposite.

### Methodological considerations

The choice of organising the data per year in the analysis was to some extent pragmatic. Dates are available in the data for all contacts with the different types of services. However, performing a full repeated measures survival analysis would be very complex due to the number of time-dependent variables involved, and also with uncertain gains given the exploratory focus of the paper. There could also be additional clustering by GPs in the data, but this is indirectly taken into account by some of the GP characteristics (contact rates GP to other providers, list length) which are constant or close to constant over time and have unique values for most GPs.

### Strengths and limitations

The main strength of the paper is the extensive data. Covering several outpatient services, in addition to inpatient, comorbidity, socioeconomic and–demographic information makes a unique data set. The fact that registration of healthcare activity in KUHR and NPR is necessary for reimbursement in primary and secondary care provides an incentive for completeness in the registries. The population-based nature of the data means that a broad range of COPD patients are included; both new and old cases, presenting mild and severe symptoms.

As previously mentioned, a limitation of the study is the lack of clinical COPD severity information for patients in the sample. Medication data that could potentially indicate severity is also missing. This is a common challenge in administrative data, and it would be of interest to estimate the association between COPD severity and later number of COPD hospital admissions. We may only assume that the frequency and type of healthcare contacts with a respiratory main diagnosis to some extent reflect the COPD severity, but we are not able to test this assumption. Another limitation is the use of primary care diagnosis as inclusion criteria and when counting the number of contacts for each service. As most contacts in primary care includes attention to more than one diagnosis, there could be some degree of arbitrariness to which diagnosis is registered in the data and the timing of the first COPD diagnosis, when several conditions are present in the patient. This is also the reason why services are split into respiratory vs. non-respiratory diagnoses in the analysis, instead of using the exact codes for COPD versus non-respiratory diagnoses. On the other hand, since the follow-up period extends over several years for most patients in the sample, comorbidities should be captured given the high frequency of health service contacts.

With respect to generalisability, using data from only two municipalities has both advantages and disadvantages. It reduces heterogeneity related to organisational and distance factors in accessing primary and specialist care. However, as the paper primarily focuses on identifying associations rather than causality, one could similarly argue that not using data from all of Norway is a disadvantage, as it prevents the assessment of whether the associations differ across the country.

## Conclusions

We found that increasing numbers of GP contacts and outpatient contacts with hospital were highly associated with future hospital admissions for COPD. Hence, at least in the absence of severity information, data on previous outpatient care seems important to include in analyses of interventions aiming to reduce hospital admissions for COPD patients. For readmissions, the value of including data on previous outpatient use was less evident. Findings further indicated that variables related to continuity, timeliness and interaction in the care of COPD patients were associated with fewer next-year hospital admissions. However, these findings should be approached with caution due to the observational nature of the data and the limited detail on the content in these variables.

### Electronic supplementary material

Below is the link to the electronic supplementary material.

Supplementary Material 1

## Data Availability

The datasets used in the current study are based on national registries and are not publicly available. Access to pseudonymised data from the national registries are only granted through application to the Norwegian Centre for Research Data and Regional Committees for Medical and Health Research Ethics.

## Declarations

### Disclaimer

Data from the Norwegian Patient Registry, Statistics Norway, and the Cause of Death Registry are used in the project. The interpretation and reporting of these data are the sole responsibility of the authors, and no endorsement by the registry owners is intended, nor should it be inferred.

## Abbreviations

COPD: Chronic Obstructive Pulmonary Disease
ICD-10: International Classification of Diseases, 10th revision
ICPC-2: International Classification of Primary Care, 2nd version (ICPC-2)
KUHR: Database for the Control and Reimbursement of Health Care claims
MEPJ: Municipality electronic patient journal
NPR: Norwegian Patient Registry
RGPR: Regular General Practitioner Registry
