# Supplementary material for: Associations between outpatient care and later hospital admissions for patients with chronic obstructive pulmonary disease - a registry study from Norway
Source: BMC Health Serv Res. 2024 Apr 22;24:500. doi: 10.1186/s12913-024-10975-4 (PMC11036724; doi:10.1186/s12913-024-10975-4)
Supplement: Supplementary file 1 — Supplementary Material 1 [file 12913_2024_10975_MOESM1_ESM.pdf]

Supplementary material

Table S1: Definitions of comorbidities by ICD-10 and ICPC-2 codes.

**Variable:**	**ICD-10:**	**ICPC-2:**	**Sample prevalence**
Alcoholism	F10-F17	P15-P16	10%
Anemia	D5*-D6*	N80-N82	14%
Cancer (except lung cancer)	All C except C3	A79, B72-B74, D74-D77, F74, H75, K72, L71, N74, R84-R85, S77, T71, U75-U77, X75-X77, Y77-Y78	24%
Cardiovascular disease	I20, I25, I48, I70-79	K74-K76, K78-80, K92	41%
Dementia	F00-F03, G30	P70	6%
Depression and anxiety	F32-F34, F40-F41	P74, P76	23%
Diabetes	E10-E14	T89-T90	17%
Heart failure	I50	K77	18%
Hypertension	I10-I15	K86-K87	46%
Kidney disease or failure	N18-19	U88, U99	10%
Lung cancer	C3*	R84-R85	5%
Mental disorders	F20-F31	P72-P73, P75, P77, P79-P80, P82, P98-P99	8%
Myocardial infarction (incl. angina pectoris)	I20-I22	K74-K75	19%
Obesity	E65-E66	T82	5%
Osteoporosis	M81	L95	15%
Stroke	I60-I64, G45	K89-K91	14%
Underweight	E40-E46	T05, T08, T11, T28, T91, T99	31%

Specification of the regression models for next-year hospital admissions and 90-day readmissions

A standard specification of the negative binomial regression model was used in all analyses, clustered by patient id-number to take into account repeated observations over time. For patient *i* in follow-up year/90-day period *t*, the equations were given by:

$$Log(Hosp\_Ad_{i,t+1}) = \beta_0 + \beta_1 Hosp\_Ad_{i,t} + \sum_{j=2}^{32} \beta_j HS\_Comb_{i,t} + \sum_{j=33}^{34} \beta_j HS\_resp_{i,t} + \sum_{j=35}^{41} \beta_j HS\_Nresp_{i,t} + \sum_{j=42}^{49} \beta_j Care\_Ind_{i,t} + \sum_{j=50}^{55} \beta_j GP\_Char_{i,t} + \sum_{j=56}^{76} \beta_j Oth\_Health_{i,t} + \sum_{j=77}^{84} \beta_j SED_{i,t} + \varepsilon_{i,t}$$

$$Log(Readm_{90_{i,t}}) = \beta_0 + \sum_{j=1}^{8} \beta_j HS\_resp_{i,t-1} + \sum_{j=9}^{15} \beta_j HS\_Nresp_{i,t-1} + \sum_{j=16}^{22} \beta_j Care\_Ind_{i,t-1} + \sum_{j=23}^{28} \beta_j GP\_Char_{i,t-1} + \sum_{j=29}^{49} \beta_j Oth\_Health_{i,t-1} + \sum_{j=50}^{57} \beta_j SED_{i,t-1} + \varepsilon_{i,t}$$

The independent variables were as follows:

Variables	Description	Outcome
	**Outpatient care, respiratory diagnoses**	
Hosp_Ad, number of COPD hospital admissions	Number of COPD hospital admissions during year	Hospital admissions
HS_Comb, combinations of outpatient respiratory care	Combinations of GP, contract specialist, outpatient hospital and physiotherapist use during year, in 32 categories (no use is reference category)	Hospital admissions
HS_resp, other variables for outpatient respiratory care	For both outcomes these included number of emergency room contacts and rehabilitation stays during the year (2 variables). For 90-day readmissions they also included GP contacts in 4 categories, and indicators of any contract specialist, outpatient hospital and physiotherapist use during the 90 days prior (6 independent variables, no combinations due to smaller readmission sample)	Hospital admissions, 90-day readmissions
	**Healthcare, non-respiratory diagnoses**	
HS_{Nresp}, variables for non-respiratory care	These included number of GP, contract specialist, physiotherapy, emergency room and outpatient hospital contacts, as well as rehabilitation stays and hospital admissions. All continuous as total per year or 90 days prior. 7 variables in total.	Hospital admissions, 90-day readmissions
	**Outpatient care indicators**	
$Care\_Ind$, variables capturing specific outpatient care	These included yearly continuity of care index and number of home visits by GP (continuous), indicators for spirometry and care interaction fees during the year, and variables capturing follow-up by GP/rehabilitation within 30 days after discharge from COPD hospital admissions (categorical). 8 variables in total for hospital admissions, 7 for 90-day readmissions.	Hospital admissions, 90-day readmissions
	**Characteristics of the regular GP**	

GP_Char, variables for GP characteristics	These included indicators of the regular GP being a specialist in general practice, practicing in a location shared with other GPs (categorical), and number of individuals on the GP's patient list, contract specialist, physiotherapy and outpatient hospital contacts per year per listed patient in sample (continuous). 6 variables in total.	Hospital admissions, 90-day readmissions
	**Other health factors**	
Oth_Health, variables for other health factors	These included yearly indicators for home nursing, nursing home, death and 17 comorbidities, as well as need-score for assistance in daily activities (continuous). 21 variables in total	Hospital admissions, 90-day readmissions
	**Socioeconomics and -demographics**	
SED, variables for socioeconomics and -demographics	These included age and gross income (continuous), as well as gender, marital status when entering sample, highest attained education and indicator of permanent disability pension prior to or during follow-up (all categorical). 8 variables in total.	Hospital admissions, 90-day readmissions

All independent variables were treated as time-dependent in the analyses, except gender, marital status when entering sample, highest attained education and the indicator of permanent disability pension prior to or during follow-up. For additional information on variables definitions and operalisation, see Methods section in the main text.

Table S2: Regression results for the remaining variables in the model, not shown in Table 3. IRR=Incidence rate ratio, 95%-CI=95% confidence interval. In the analysis of 90-day readmissions, health service use were entered as continuous variables in the model due to fewer observations. *=significant at 5%-level.

Outcome:	Next-year COPD hospital admissions (n=148,128)	90-day readmissions (n=18,372 index hospital admissions)
**Independent variable:**	**IRR (95%-CI)**	**IRR (95%-CI)**
**Health service use, respiratory diagnoses**		
GP contacts: 1-2 vs. none	See Fig 1	1.30 (1.14-1.48)*
3-4 vs. none		1.40 (1.19-1.64)*
5 or more vs. none		1.37 (1.17-1.61)*
Contract specialist contacts	See Fig. 1	0.78 (0.61-0.99)*
Physiotherapy contacts	See Fig. 1	0.92 (0.73-1.16)
Outpatient hospital contacts	See Fig. 1	1.08 (0.94-1.24)
Municipal emergency room contacts	1.06 (1.04-1.08)*	1.19 (1.06-1.33)*
Rehabilitation stays	1.01 (1.00-1.03)*	0.93 (0.84-1.03)
COPD hospital admissions	1.27 (1.25-1.30)*	N/A
**Health service use, non-respiratory diagnoses**		
GP contacts	1.01 (1.00-1.01)*	1.01 (0.99-1.02)
Contract specialist contacts	0.98 (0.97-0.99)*	0.91 (0.85-0.97)*
Physiotherapy contacts	0.99 (0.99-1.00)*	0.99 (0.96-1.02)
Municipal emergency room contacts	0.99 (0.98-0.99)*	1.00 (0.97-1.04)
Outpatient hospital contacts	1.00 (0.99-1.00)	1.01 (0.99-1.03)
Rehabilitation stays	0.97 (0.96-0.99)*	0.94 (0.86-1.04)
Hospital admissions	1.01 (1.01-1.02)*	1.17 (1.05-1.29)*
**Comorbidities**		
Cancer (except lung cancer)	0.93 (0.87-0.99)*	0.87 (0.74-1.02)
Cardiovascular disease	1.04 (0.98-1.09)	1.01 (0.89-1.15)
Dementia	0.67 (0.58-0.76)*	0.80 (0.56-1.15)
Heart failure	1.09 (1.02-1.17)*	1.05 (0.91-1.20)
Hypertension	0.88 (0.84-0.93)*	1.14 (1.00-1.30)
Kidney disease or failure	1.00 (0.91-1.10)	1.10 (0.90-1.34)
Myocardial infarction	1.10 (1.02-1.18)*	1.00 (0.82-1.21)
Obesity	1.04 (0.92-1.19)	0.86 (0.61-1.22)
Stroke	1.00 (0.91-1.10)	1.19 (0.96-1.48)
**Long-term care and end of life**		
Activities of daily living sum-score (per 10 points)	1.10 (1.08-1.11)*	1.01 (0.97-1.06)
Home nursing	1.12 (1.04-1.20)*	1.03 (0.88-1.21)
Nursing home	0.49 (0.40-0.60)*	0.89 (0.66-1.19)
Next-year death	1.46 (1.36-1.57)*	1.29 (1.08-1.55)*
**Sociodemographics**		
Age	1.03 (1.03-1.04)*	1.01 (1.00-1.02)
Female gender	0.87 (0.83-0.92)*	0.89 (0.78-1.02)
Marital status widow/widower vs. married	1.21 (1.13-1.29)*	1.03 (0.87-1.21)
Marital status not married/divorced vs. married	1.04 (0.99-1.11)	1.03 (0.91-1.17)

Figure S1: Relative change (incidence rate ratio and 95% confidence interval) in next-year COPD admissions for combinations of current year health service use, % of total and number of observations within each combination.

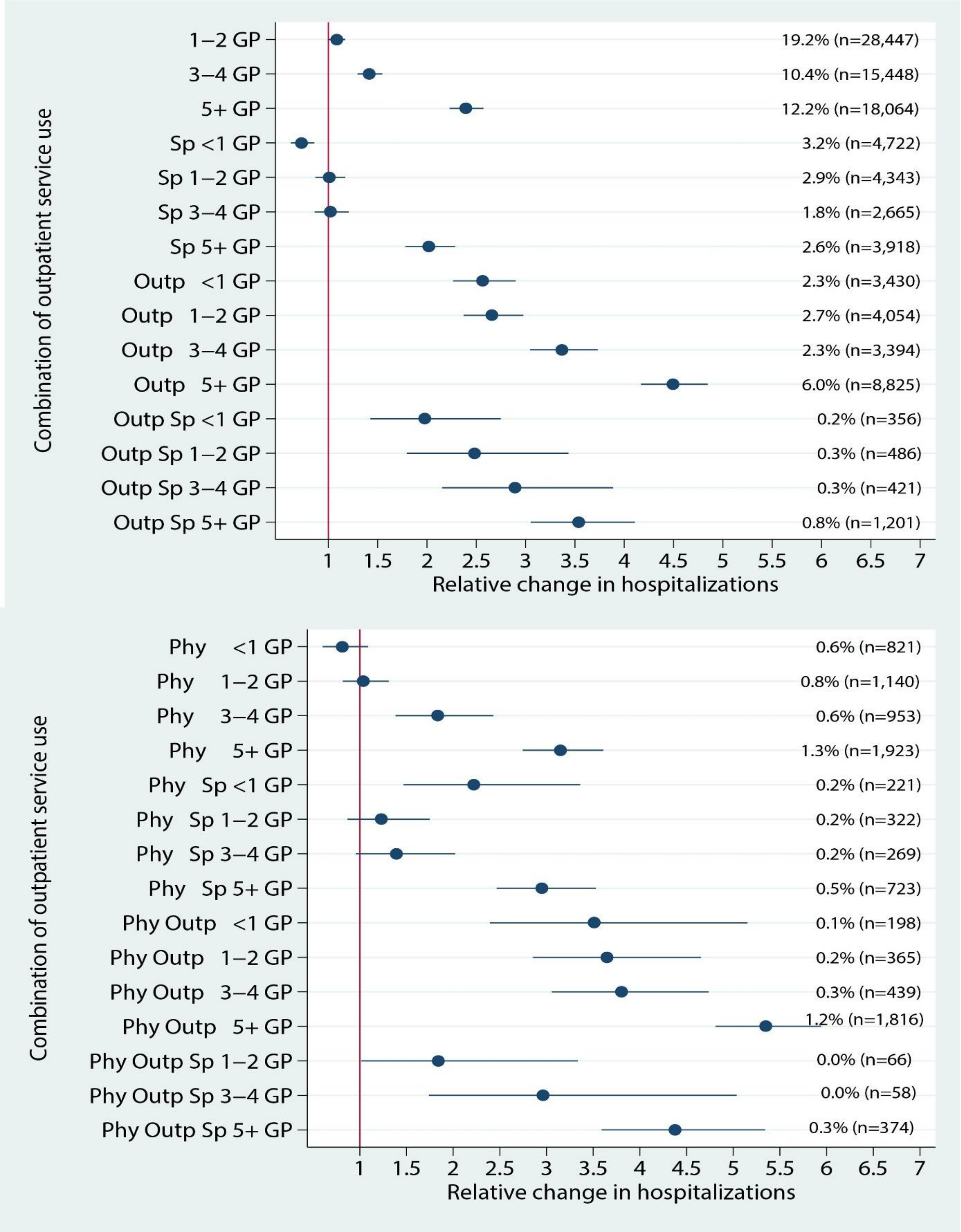

Note: Adjusted for current year COPD hospital admissions. The reference category is no contacts for respiratory diagnoses (26% of total, n=38,637 observations). The category no GP contact and use of all other services is omitted due to few observations (n=29). N=148,128 observations from 24,074 patients. GP=general practitioner, Sp=contract specialist contact, Outp=outpatient contact with hospital, Phy=physiotherapy contact.

Table S3: Simple regression results for the outpatient care indicators, characteristics of the regular GP and selected socioeconomic and comorbidity variables. Analyses of next-year COPD hospital admissions are adjusted for current year COPD admissions. IRR=Incidence rate ratio, 95%-CI=95% confidence interval. *=significant at 5%-level.

**Outcome:**	**Next-year COPD hospital admissions (n=148,128)**	**90-day readmissions (n=18,372 index hospital admissions)**
**Independent variable**:	**IRR (95%-CI)**	**IRR (95%-CI)**
**Care indicators**		
Spirometry performed at GP or contract specialist	1.05 (1.01-1.11)*	1.08 (0.94-1.24)
Continuity within GP care	0.93 (0.87-1.00)	0.92 (0.78-1.10)
Rehabilitation within 30 days vs. later after COPD hospital admission	0.90 (0.77-1.06)	0.86 (0.69-1.06)
No hospital admission/rehabilitation vs. later rehabilitation after COPD hospital admission	0.71 (0.62-0.83)*	0.43 (0.37-0.50)*
Follow-up by GP within 30 days vs. no/later follow-up after COPD hospital admission	1.20 (1.13-1.28)*	1.13 (1.00-1.27)*
No hospital admissions vs. no/later follow-up by GP after COPD hospital admission	0.34 (0.32-0.36)*	N/A
Care interaction fees	1.18 (1.06-1.30)*	1.18 (1.03-1.36)*
Home visits by GP or physiotherapist	1.02 (1.01-1.03)*	1.01 (1.00-1.02)*
**Characteristics of the regular GP**		
GP specialist	0.98 (0.92-1.04)	1.03 (0.94-1.13)
GP group practice	0.91 (0.80-1.02)	1.03 (0.78-1.36)
List length per 100 patients	0.99 (0.98-1.00)	1.00 (0.99-1.02)
Contact rate GP and contract specialist, respiratory diagnoses	1.08 (1.03-1.14)*	1.03 (0.94-1.13)
Contact rate GP and physiotherapist, respiratory diagnoses	1.01 (1.00-1.01)*	1.00 (0.99-1.01)
Contact rate GP and outpatient hospital, respiratory diagnoses	1.12 (1.01-1.24)*	1.01 (0.98-1.04)
**Comorbidities**		
Alcoholism	1.47 (1.33-1.62)*	1.30 (1.03-1.63)*
Anemia	1.44 (1.31-1.57)*	1.36 (1.13-1.64)*
Depression	1.31 (1.23-1.41)*	1.06 (0.93-1.21)
Diabetes	0.98 (0.91-1.05)*	0.96 (0.82-1.13)
Lung cancer	1.80 (1.61-2.03)*	1.28 (1.03-1.59)*
Mental disorders	1.05 (0.94-1.18)	0.97 (0.75-1.27)
Osteoporosis	1.46 (1.34-1.59)*	1.16 (0.99-1.37)
Underweight	1.31 (1.23-1.40)*	1.30 (1.12-1.52)*
**Socioeconomics**		
Gross income (per 100,000 NOK)	0.90 (0.87-0.93)*	0.98 (0.94-1.02)
Education: Secondary vs. primary	0.83 (0.79-0.87)*	0.94 (0.84-1.05)
College/university vs. primary	0.55 (0.51-0.59)*	0.89 (0.74-1.06)
Permanent disability pension	1.12 (1.07-1.17)*	0.88 (0.79-0.98)*

Table S4: Simple regression results for the remaining variables in the model, not shown in Table S3. Analyses of next-year COPD hospital admissions are adjusted for current year COPD admissions. IRR=Incidence rate ratio, 95%-CI=95% confidence interval. *=significant at 5%-level.

**Outcome:**	**Next-year COPD hospital admissions (n=148,128)**	**90-day readmissions (n=18,372 index hospital admissions)**
**Independent variable:**	**IRR (95%-CI)**	**IRR (95%-CI)**
**Health service use, respiratory diagnoses**		
GP contacts: 1-2 vs. none	See Fig 1	1.24 (1.09-1.41)*
3-4 vs. none		1.37 (1.18-1.59)*
5 or more vs. none		1.50 (1.31-1.72)*
Contract specialist contacts	See Fig 1	0.70 (0.56-0.87)*
Physiotherapy contacts	See Fig 1	0.91 (0.73-1.13)
Outpatient hospital contacts	See Fig 1	1.17 (1.02-1.33)*
Municipal emergency room contacts	1.24 (1.20-1.27)*	1.26 (1.14-1.38)*
Rehabilitation stays	1.06 (1.05-1.08)*	0.93 (0.86-1.02)
**Health service use, non-respiratory diagnoses**		
GP contacts	1.02 (1.01-1.02)*	1.02 (1.01-1.03)*
Contract specialist contacts	0.99 (0.99-1.00)*	0.93 (0.88-0.99)*
Physiotherapy contacts	0.99 (0.99-0.99)*	0.98 (0.96-1.00)
Municipal emergency room contacts	1.04 (1.03-1.06)*	1.05 (1.02-1.08)*
Outpatient hospital contacts	1.01 (1.01-1.01)*	1.02 (1.00-1.03)*
Rehabilitation stays	1.01 (1.00-1.02)*	1.03 (0.98-1.07)
Hospital admissions	1.10 (1.07-1.13)*	1.28 (1.15-1.43)*
**Comorbidities**		
Cancer (except lung cancer)	1.11 (1.04-1.19)*	0.98 (0.85-1.13)
Cardiovascular disease	1.43 (1.37-1.50)*	1.19 (1.07-1.32)*
Dementia	1.11 (0.97-1.28)	0.97 (0.71-1.34)
Heart failure	1.87 (1.75-1.99)*	1.29 (1.14-1.47)*
Hypertension	0.97 (0.92-1.02)	1.23 (1.09-1.40)*
Kidney disease or failure	1.45 (1.33-1.58)	1.41 (1.19-1.68)*
Myocardial infarction	1.47 (1.37-1.58)*	1.20 (1.01-1.43)*
Obesity	1.02 (0.88-1.17)	0.88 (0.62-1.23)
Stroke	1.22 (1.11-1.34)*	1.42 (1.15-1.75)*
**Long-term care and end of life**		
Activities of daily living sum-score (per 10 points)	1.22 (1.21-1.24)*	1.08 (1.05-1.11)*
Home nursing	1.96 (1.84-2.09)*	1.36 (1.22-1.51)*
Nursing home	0.82 (0.69-0.97)*	0.90 (0.70-1.16)
Next-year death	2.42 (2.27-2.59)*	1.40 (1.19-1.64)*
**Sociodemographics**		
Age	1.03 (1.03-1.04)*	1.01 (1.01-1.02)*
Female gender	1.08 (1.03-1.13)*	0.92 (0.83-1.02)
Marital status widow/widower vs. married	1.66 (1.55-1.76)*	1.11 (0.96-1.29)
Marital status not married/divorced vs. married	1.01 (0.96-1.07)	1.00 (0.89-1.12)
